# *Tunabio*: biological traits of tropical tuna and bycatch species caught by purse seine fisheries in the Western Indian and Eastern Central Atlantic Oceans

**DOI:** 10.3897/BDJ.10.e85938

**Published:** 2022-07-22

**Authors:** Aurelie M Guillou, Nathalie Bodin, Emmanuel Chassot, Antoine Duparc, Théotime Fily, Philippe S Sabarros, Mathieu Depetris, Monin J Amandè, Juliette Lucas, Emilie Augustin, N’guessan C Diaha, Laurent Floch, Julien Barde, Pedro J Pascual-Alayón, José Carlos Báez, Pascal Cauquil, Karine Briand, Julien Lebranchu

**Affiliations:** 1 MARBEC, Univ Montpellier, CNRS, Ifremer, IRD, Sète, France MARBEC, Univ Montpellier, CNRS, Ifremer, IRD Sète France; 2 Sustainable Ocean Seychelles (SOS), BeauBelle, Mahé, Seychelles Sustainable Ocean Seychelles (SOS) BeauBelle, Mahé Seychelles; 3 African Marine Expertises (Amexpert), Abidjan, Cote d'Ivoire African Marine Expertises (Amexpert) Abidjan Cote d'Ivoire; 4 Seychelles Fishing Authority (SFA), Victoria, Seychelles Seychelles Fishing Authority (SFA) Victoria Seychelles; 5 Centre de Recherches Océanologiques (CRO), Abidjan, Cote d'Ivoire Centre de Recherches Océanologiques (CRO) Abidjan Cote d'Ivoire; 6 Instituto Español de Oceanografía (IEO), Tenerife, Spain Instituto Español de Oceanografía (IEO) Tenerife Spain; 7 Instituto Español de Oceanografía (IEO), Malaga, Spain Instituto Español de Oceanografía (IEO) Malaga Spain

**Keywords:** tropical tunas, bycatch fish, purse seine, length-length and length-weight relationships, sex-ratio, maturity stage, gonad weight, diet

## Abstract

**Background:**

Along with the development of the tropical tuna purse-seine fishery from the 1960s in the Atlantic Ocean and from the 1980s in the Indian Ocean, many projects and studies have been conducted to improve knowledge about the biology, migrations and dynamics of the stocks of target and non-target (i.e. bycatch) species taken in these fisheries. Since the 2000s, the European Union (EU) has been supporting Member States in the collection of biological data on species caught by their purse seine and pole and line fisheries, thus making it possible to have a long-term series of data. Biological data have never been saved by the different tuna commissions, unlike the catches by species and sizes by areas and periods. However, these data are essential to monitor the status of the fisheries and fuel the assessment models used by the tuna Regional Fisheries Management Organisations (tRFMOs) for the sustainable management and conservation of the fish stocks under their mandate.

**New information:**

We combined historical (1974-1999) and current (2003-2020) datasets on the biology of tropical tunas and bycatch fish caught by large-scale purse seiners in the Eastern Atlantic Ocean (EAO) and Western Indian Ocean (WIO). The resulting *Tunabio* database is presented in the present data paper and contains all available morphometric and biological data collected on more than 80,000 fish individuals.

## Introduction

The tropical tuna purse seine fishery has significantly increased in the Eastern Atlantic Ocean (EAO) and Western Indian Ocean (WIO) since its development in the 1960s and 1980s, respectively (Fonteneau and Marcille 1988, Báez et al. 2020). In both oceans, this fishery is dominated by the EU purse seine fleet, in particular under the Spanish and French flags (Chassot et al. 2013, Pascual-Alayón et al. 2019). In 2020, this fleet was composed of about eighty large-scale purse seiners in the EAO and WIO (Báez et al. 2020, Floch et al. 2020). Vessels target the three principal commercial tropical tuna species: yellowfin tuna (*Thunnusalbacares*, YFT), skipjack tuna (*Katsuwonuspelamis*, SKJ) and bigeye tuna (*Thunnusobesus*, BET). In these both areas, the EU annual catch during the period 2010-2019 represented on average about 30% of the total catch of tropical tuna (ICCAT 2020, IOTC 2021). A large number of bycatch species (non-targeted species) is also caught with purse seine, such as common dolphinfish (*Coryphaenahippurus*), frigate and bullet tunas (*Auxisthazard* and *Auxisrochei*), little tunny (*Euthynnusalletteratus*), rough triggerfish (*Canthidermismaculata*) and many other species (Amandè et al. 2011, Amandè et al. 2012, Ruiz et al. 2018).

Considering the economic and nutritional importance of tuna, the need for knowledge on these species quickly arose to support the monitoring of the fisheries and the development and implementation of some stock management measures by the tRFMOs. From the 1960s and until 2000, data on the biology of species were collected throughout several scientific projects focusing on the principal market tuna species. The first studies on biological traits of tropical tuna mainly focused on migrations and biometrics of yellowfin tuna (Postel 1955). At first, morphometric data were sampled (Rossignol 1968, Caverivière 1976, Cayré and Laloë 1986, Gaertner et al. 1999) in order to estimate the number of individuals by size class caught during a trip or to test for differences in length-weight relationships between seasons or areas. They were collected on the ships during landings or in the canneries. On purse seiners, only the predorsal length can be measured on large tunas. Other measurements, such as weight and fork length, taken in the laboratory and in the cannery, are therefore essential. These measurements are used to convert the predorsal length and to estimate the catch-at-size of large tunas and the catch composition. Collection was further supplemented with reproductive data (sex, macro-maturity of gonads...) (Albaret 1976, Stéquert 1976, Cayré et al. 1988, Karpinski and Hallier 1988, Hassani and Stéquert 1990, Bard and Capisano 1991) and later on with dietary data (Ménard et al. 2000, Potier et al. 2004). These research and data collection projects aimed to describe the biological characteristics of fish stocks (length-weight relationships between gender, sex-ratio at size and age at sexual maturity), their evolution and the effects of fisheries. These data were also used as inputs for length-based and age-structured assessment models as well as to investigate the seasonal and interannual changes in the condition status of individuals, the variability of the diet linked to their environment and the cycle and periods of reproduction (Albaret et al. 1976, Hassani and Stéquert 1990, Sardenne et al. 2016).

Since 2000, the Common Fisheries Policy (CFP) has been governing the collection, administration and use of fishery data with the aim of marine resource assessment and management*^1^. The CFP has been funded through the European Maritime and Fisheries Funds and managed by the Department of Marine Fisheries and Aquaculture for France. The EU Data Collection Framework (DCF - Reg 199/2008 and 665/2008 EU-2017/1004) has been regulating the collection of fisheries data since 2000. Several successive multi-annual programmes,*^2^ such as the EU Multi-Annual Programme 2017-2019 (EUMAP 2016/1251), have been implemented by each Member State in their National Work Plan*^3^. Each country has the obligation to monitor its fleet to ensure compliance with the decisions and rules taken in the various regional fisheries bodies, such as the International Commission for the Conservation of Atlantic Tunas (ICCAT) and the Indian Ocean Tuna Commission (IOTC). Collected data are diverse and cover all the activities carried out by a vessel: catches of target and non-target species, fishing effort, biological data etc. (DPMA 2017, Bach et al. 2018). These data are used to provide scientific advice and recommendations. The Exploited Tropical Pelagic Ecosystems Observatory*^4^ (Ob7) of the French National Research Institute for Sustainable Development*^5^ (IRD, previously named Office of Scientific and Technical Research Overseas, ORSTOM, until 1998) is in charge of the data collection regarding the French tropical purse seine fleet in the Atlantic and Indian Oceans.

This document presents the data collected by IRD in the Atlantic and Indian Oceans since the 1970s. The different datasets are stored in a database called *Tunabio*.

## General description

### Purpose

The key goal of *Tunabio* is to merge and make available biological datasets of purse seine tropical tuna and bycatch species from the EAO and WIO into a single open-access database. The *Tunabio* database regroups a total of nine datasets (Table 1): six datasets were collected as part of historical projects carried out in the EAO (ALBARET_PHD, IRD_1983-1988 and RONDEUR) and in the WIO (BIOM_BET, BIOMCO and IOT_STOMACHS) and the last two datasets were collected as part of long-term ongoing projects (RTP_DEBARQUEMENT in the Atlantic Ocean and DCF/EUMAP in both oceans).

## Sampling methods

### Sampling description

Projects having different objectives, the sampling approaches varying from one to another. As a consequence, data collection took place on board the purse seine vessels during the fishing trips, at the port during fish unloading or after landing at the tuna processing factories (canneries) or at the partners’ research laboratories. The detailed sampling description can be found in the different project documentation (see Table 1 for references).

### Quality control

Data stored in *Tunabio* database are systematically checked for integrity. Consistency, validation and formatting controls are carried out when entering the data. Errors can be made at the time of transcription and data entry. Data are, therefore, controlled with charts, comparison of values or by checking the timeline for the dates. A correction is made on the basis of the physical data entry sheets on which the data are recorded during the sampling.

### Step description

**Figure 1.**
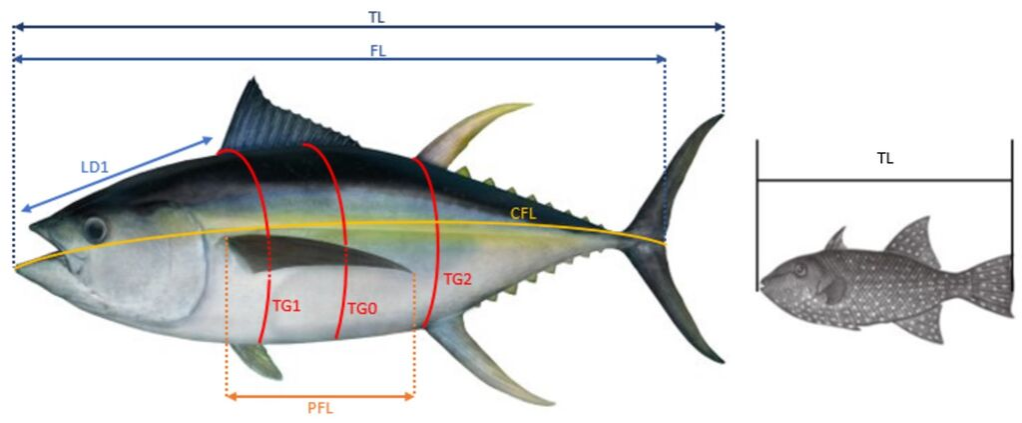
Morphometric measurements on a fish (example on a tuna on the left and a triggerfish on the right). TL: total length, FL: fork length, LD1: predorsal length, CFL: curve fork length, TG0: middle thorax girth, TG1: first thorax girth, TG2: second thorax girth, PFL: pectoral fin length. The measurement of the TL depends on the stiffness of the tail.

**Biological measurements**: The different types of morphometric measurements taken on tropical tunas and bycatch fish are shown in Fig. 1 and further described in the **Data resources section**. Information on the measurement device (calliper, tape measure or fish ruler) was also recorded when possible. In addition to the morphometric measurements, the sex (S), the weight of the fish (whole fish weight - WFW and gutted fish weight - GFW) and the weight of reproductive and digestive organs (gonads weight - GTW, liver weight - LW, full and empty stomach weight - FSW and ESW) were recorded for tropical tunas and bycatch fish. The projects studying the stage of gonad development (macro maturity - MM) did not use the same scales. A modification was made in Tunabio to obtain three stages: immature, developing and spawning. Finally, the stomach contents were analysed by sorting and identifying the main taxonomic groups of prey present.

**Traceability**: The traceability corresponds to the ability to accurately and precisely determine the origin of a fish sampled, i.e. the geographic location and date when the fish was caught at sea. This also includes the vessel and trip identifiers. Traceability is dependent on the vessel configuration and sampling constraints (e.g. access to the vessel, configuration of the wells, sorting occurring at unloading). At sea, the catch from a fishing set can be stored in different wells on board and several fishing sets can be stored in the same well, making the catch date and location lost or inaccurate. Information on fishing operations are collected from the logbook and well plan of the trip associated.

**Figure 2.**
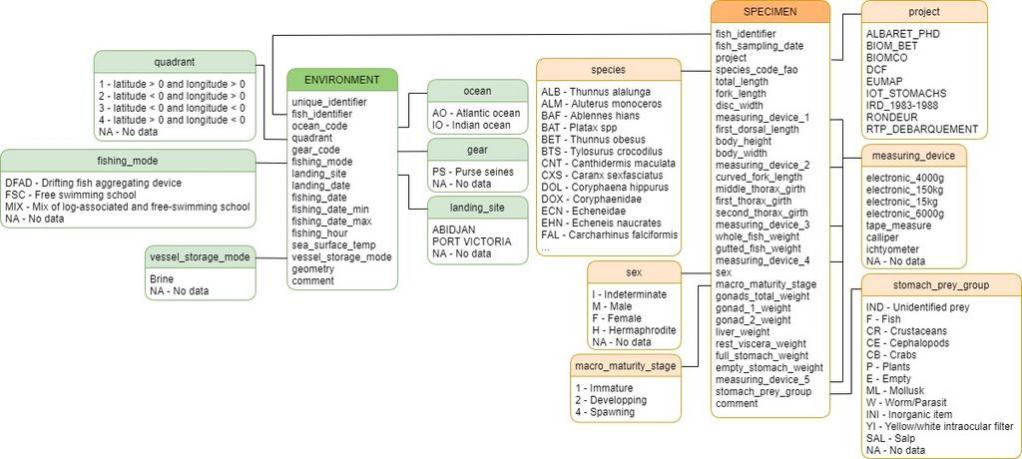
Structure of the Tunabio database, based on the two record tables (ENVIRONMENT and SPECIMEN) and the twelve sheets of reference lists.

**Database description and input particularity**: *Tunabio* includes the ‘METADATA’ sheet that describes the variables used in the data sheets ‘ENVIRONMENT’ and ‘SPECIMEN’ and 12 sheets of code lists used as integrity check and validation (see **Quality control section**) to complete the two previous sheets (Fig. 2). Reference lists are described in Table 2.

Logbooks and well plan data are stored in the ‘ENVIRONMENT’ sheet. The biological data collected are entered in the ‘SPECIMEN’ sheet. For biological data, each identifier is unique in the ‘SPECIMEN’ sheet. Due to the multiple possibilities of dates and fishing positions, a given fish identifier can be repeated in the ‘ENVIRONMENT’ sheet and each position is entered as POINT (single location). If the well is unknown, all trip positions are entered as MULTIPOINT (multiple locations). For this case, a given fish identifier would only appear once. The fishing positions extracted from the logbooks are in the “degree minutes” format. When entering these locations, they are converted into Well-Known Text format (WKT).

A recurring problem concerns the well plan for vessels which have a front or central well. There may be a discrepancy between the numbering of the wells on the well plan in the logbook and the numbering assigned by the canneries (which may not always be consistent). A well number assigned could, therefore, match two wells due to the offset. In such a case, the information of the two wells was entered in order not to miss any fishing information. Another problem concerns the fish arriving by freighter and container that come from an ocean other than the one where the final landing occurs: the only information obtained are those given by the factories and they cannot be verified in such cases.

## Geographic coverage

### Description

**Figure 3.**
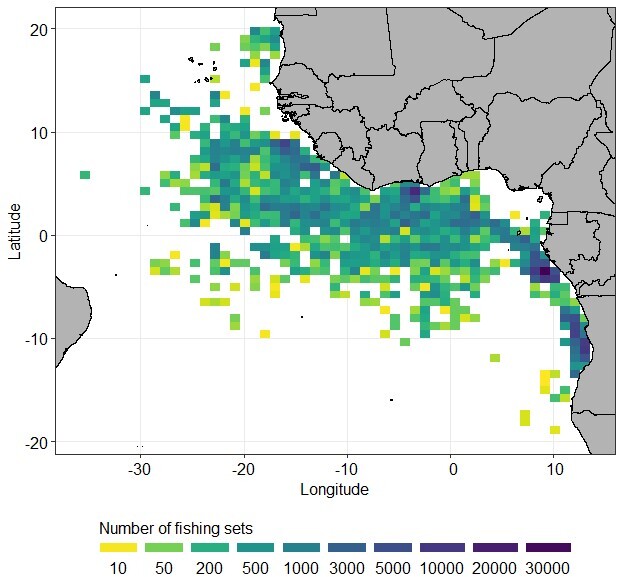
Densities of fishing sets (number per 1° square) of the tropical tunas and bycatch sampled in the Eastern Atlantic Ocean during 1983-2020.

In the EAO, the fishing zone extends from Mauritania (20° North) to Angola (20° South) (Fig. 3). The western maximum limit is at 35° West. Amongst the 36,019 fish sampled, there are 2,407 for which the fishing position is unknown. Fifteen of them were caught in the Indian Ocean and transported and sampled in Abidjan (Ivory Coast).

**Figure 4.**
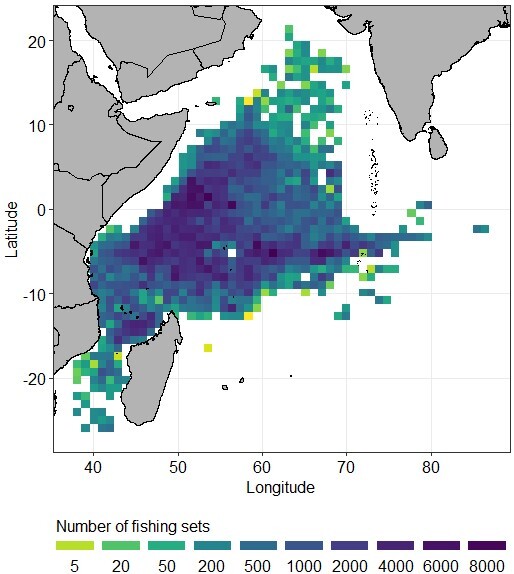
Densities of fishing sets (number per 1° square) of the tropical tunas and bycatch fish species sampled in the Western Indian Ocean during 1987-2020.

In the WIO, the fishing zone extends from the Arabian Sea (20° North) to the south of Madagascar (25° South) (Fig. 4). The western and eastern maximum limits are approximately 42° East and 87° East. Amongst the 44,292 fish sampled, there are 1,279 fish for which the fishing position is unknown.

## Taxonomic coverage

### Description

*Tunabio* currently contains 80,311 fish individuals (36,019 unloaded at the port of Abidjan, Ivory Coast, Atlantic Ocean and 44,292 unloaded at the port of Victoria, Seychelles, Indian Ocean) divided into 32 taxonomic groups including 27 at the species level (Table 3).

## Temporal coverage

**Data range:** 1974-1-01 – 2020-12-31; 1987-1-01 – 2020-12-31.

### Notes

*Tunabio* covers the periods 1974-2020 and 1987-2020 in the EAO and WIO, respectively (Guillou et al. 2022). The largest dataset concerns yellowfin tuna which was regularly sampled throughout those periods. Sampling of bigeye and skipjack tunas started in 1998 in the EAO; it started in 1988 and 2014, respectively, in the WIO. Data collection on bycatch fish species started in 2016 in the EAO and in 2009 in the WIO.

## Usage licence

### Usage licence

Creative Commons Public Domain Waiver (CC-Zero)

## Data resources

### Data package title

Tunabio_1974_2020.zip

### Resource link

https://doi.org/10.17882/73500

### Number of data sets

2

### Data set 1.

#### Data set name

ENVIRONMENT

#### Data format

version 1974-2020

#### Download URL

https://doi.org/10.17882/73500

#### Description

The dataset includes the fishing data (e.g. fishing location, date/time, gear) retrieved from the purse-seine logbooks for each sampled fish.

**Data set 1.** 

Column label	Column description
unique_identifier	Unique identifier of fish sampled according environmental data.
fish_identifier	Identifier of fish sampled.
ocean_code	Ocean where the fish was caught. AO = Atlantic Ocean, IO = Indian Ocean.
quadrant	Geographic quadrant of the capture over the equator and the meridian of Greenwich. 1 = North East, 2 = South East, 3 = South West, 4 = North West, NA = No data.
gear_code	Fishing gear used. PS = purse seines.
fishing_mode	Aggregated fishing mode, in the case of purse seine fishing: nature of the association of the fish. DFAD = Drifting fish aggregating device, FSC = Free swimming school, MIX = Mix of log-associated and free-swimming school, NA = No data.
landing_site	Landing port of the fishing vessel (landing). For fish caught in another ocean, the landing port will be the final destination (case of fish transported by cargo ship). ABIDJAN or PORT VICTORIA.
landing_date	Arrival date of the fishing vessel in the port to unload all or a part of its catch. For fish caught in another ocean, the arrival date will be the final destination (case of fish transported by cargo ship).
fishing_date	Fish catching date. For European purse seiners, the fish might be sampled in a well and several fishing dates can be associated with the sample (mix of several fishing sets). All fishing dates of the well are noted, so the fish_identifier is duplicated (one date per row).
fishing_date_min	Several possible cases: - Case where the fishing date is known (exact date or case where the fish comes from a well with several sets): date of the first positive fishing set during the trip. - Case where the fishing date is unknown, but the landing date is known: date of the first positive fishing set during the trip. Note, a trip 'M' may not be fully landed. We find in the wells of trip M+1 fish from trip M. The first fishing set will be that of trip M and not that of trip M+1. - Case of samples taken at sea by observers: the samples are taken on a fishing set. The fishing date is known: date of the first positive fishing set during the tide. - Case of samples taken at sea by observers: the samples are over several sets/several days. The observer was not precise in noting the date (for example: from 03 Sept to 06 Sept). The fishing date is not known, but approximate: date of the first day of sampling.
fishing_date_max	Several possible cases: - Case where the fishing date is known (exact date or case where the fish comes from a well with several sets): date of the last positive fishing set during the trip. - Case where the fishing date is unknown, but the landing date is known: date of the last positive fishing set during the trip. Note, a trip 'M' may not be fully landed. We find in the wells of trip M+1 fish from trip M. The first fishing set will be that of trip M and not that of trip M+1. - Case of samples taken at sea by observers: the samples are taken on a set. The fishing date is known: date of the last positive fishing set during the tide. - Case of samples taken at sea by observers: the samples are over several sets/several days. The observer was not precise in noting the date (for example: from 03 Sept to 06 Sept). The fishing date is not known, but approximate: date of the last day of sampling.
fishing_hour	Hour at which the fishing operation took place.
sea_surface_temp	Sea surface temperature.
vessel_storage_mode	The mode of conservation of fish in the vessel. Brine = Brine immersion freezing (temperature = 18°C), NA = No data.
geometry	Fishing position of the set in WKT format. If the exact position is known, use "POINT". If the fishing set is not known, use "MULTIPOINT". In this case, enter all fishing positions.
comment	Any remarks on the fishing characteristics.

### Data set 2.

#### Data set name

SPECIMEN

#### Download URL

https://doi.org/10.17882/73500

#### Data format version

version 1974-2020

#### Description

The dataset includes the biological data collected on the tropical tunas and bycatch fish species caught by purse-seine in the EAO and WIO.

**Data set 2.** 

Column label	Column description
fish_identifier	Identifier of the fish sampled.
fish_sampling_date	Date when the fish was sampled.
project	Acronym of the research project from which the data were collected. ALBARET_PHD, DCF, EUMAP, IRD_1983-1988, RONDEUR, RTP_DEBARQUEMENT, BIOM_BET, BIOMCO, IOT_STOMACHS. See Table 1 for descriptions.
species_code_fao	Code name of the species in 3 letters following the FAO standards. ALB = Thunnusalalunga, ALM = Aluterusmonoceros, BAF = Ablenneshians, BAT = Platax spp, BET = Thunnusobesus, BTS = Tylosuruscrocodilus, CNT = Canthidermis maculate, CXS = Caranxsexfasciatus, DOL = Coryphaenahippurus, DOX = Coryphaenidae, ECN = Echeneidae, EHN = Echeneisnaucrates, FAL = Carcharhinusfalciformis, FRI = Auxisthazard, FRZ = Auxisthazard, A. rochei, GBA = Sphyraenabarracuda, KAW = Euthynnusaffinis, KYC = Kyphosuscinerascens, KYV = Kyphosusvaigiensis, LOB = Lobotessurinamensis, LTA = Euthynnusalletteratus, MSD = Decapterusmacarellus, PLS = Dasyatisviolacea, RRU = Elagatisbipinnulata, RUB = Caranxcrysos, SKJ = Katsuwonuspelamis, TRE = Caranx spp, TRI = Balistidae, USE = Uraspissecunda, WAH = Acanthocybiumsolandri, YFT = Thunnusalbacares, YTL = Seriolarivoliana.
total_length_cm	(TL) For species without caudal fork and for sharks. Projected straight distance from the tip of the longest jaw to the tip of the caudal fin. If the caudal fin is heterocercal (lobes of unequal length), the measurement is made with the larger lobe. The fin must be folded. The fish's mouth should be closed. The measure can be made on species with a caudal fork.
fork_length_cm	(FL) For species with caudal fork, but without rostrum: projected straight distance from the tip of the lower jaw to the shortest caudal ray (fork). The fish's mouth should be closed.
disc_widthc_cm	(DW) For skates. Projected straight distance between the ends of the pectoral fins.
measuring_device_1	Gear used to measure the TL, FL and DW. Tape_measure = Tape measure, calliper = Calliper, ichtyometer = Fish ruler, NA = No data.
first_dorsal_length_cm	(LD1) Projected straight distance from the upper jaw to the anterior base of the first dorsal fin.
body_height_cm	(BH) Projected straight distance of the body height where the fish is thicker.
body_width_cm	(BW) Projected straight distance of the body width where the fish is thicker.
measuring_device_2	Gear used to measure the LD1, BH and BW. Tape_measure = Tape measure, calliper = Calliper, ichtyometer = Fish ruler, NA = No data.
curved_fork_length_cm	(CFL) For species with caudal fork, but without rostrum: curved-body distance from the tip of the lower jaw to the base of the caudal fork, by the side, above the pectoral fin.
middle_thorax_girth_cm	(TG0) Circumference of the thorax where the fish is thicker.
first_thorax_girth_cm	(TG1) Circumference of the thorax just behind the pectoral and pelvic fins and in front of the first dorsal fin.
second_thorax_girth_cm	(TG2) Circumference of the thorax before the second dorsal fin and the anal fin.
measuring_device_3	Gear used to measure the CFL, TG1 and TG2. Tape_measure = Tape measure, calliper = Calliper, ichtyometer = Fish ruler, NA = No data.
whole_fish_weight_kg	(WFW) Weight of the whole fish.
gutted_fish_weight_kg	(GFW) Weight of the gutted fish.
measuring_device_4	Gear used to measure the WFW and GFW. Electronic_4000g = Electronic balance (to 4 kg), electronic_150kg = Electronic balance (to 150 kg), electronic_15kg = Electronic balance (to 15 kg), electronic_6000g = Electronic balance (to 6 kg), NA = No data.
sex	(S) Sex of the fish according to macroscopic (visual) examination.
macro_maturity_stage	(MM) Stage of the gonads according to macroscopic visual examination.
gonads_total_weight_g	(GTW) Weight of the two gonads.
gonad_1_weight_g	(G1W) Weight of the first gonad.
gonad_2_weight_g	(G2W) Weight of the second gonad.
liver_weight_g	(LW) Weight of the liver.
rest_viscera_weight_g	(RVW) Weight of other viscera (heart, oesophagus, pylorus, intestine, pyloric caeca, mesentery...) without liver, stomach and gonads.
full_stomach_weight_g	(FSW) Weight of the full stomach (stomach tissue and content).
empty_stomach_weight_g	(ESW) Weight of the stomach after removing the contents.
measuring_device_5	Gear used to measure organs. Electronic_4000g = Electronic balance (to 4 kg), electronic_150kg = Electronic balance (to 150 kg), electronic_15kg = Electronic balance (to 15 kg), electronic_6000g = Electronic balance (to 6 kg), NA = No data.
stomach_prey_group	Contents of the stomach by prey categories. If several, complete in alphabetical order, separated by ";". IND = Unidentified prey, F = Fish, CR = Crustaceans, CE = Cephalopods, CB = Crabs, PL = Plants, E = Empty, ML = Mollusc, W = Worm/Parasite, INI = Inorganic item, YI = Yellow/white intraocular filter, SAL = Salp, NA = No data.
comment	Any remark during the fish sampling.

## Figures and Tables

**Table 1.** Description of the projects that contributed to Tunabio. BET: bigeye tuna; SKJ: skipjack tuna; YFT: yellowfin tuna, TL: total length, FL: fork length, LD1: predorsal length, CFL: curve fork length, TG0-TG1-TG2: middle, first and second thorax girth, BH: body height, BW: body width, WFW: whole fish weight, GFW: gutted fish weight, S: sex, MM: macro maturity stage, GTW: gonads weight, LW: liver weight, RVW: rest viscera weight, FSW: full stomach weight, ESW: empty stomach weigh

**Ocean**	**Project name**	**Sampling period**	**Number of fish**	**Objectives**	**Data type**	**Financing**	**Lead**	**References**
Atlantic	ALBARET_PHD	1974-1975	254	Study the reproduction of the YFT in the Gulf of Guinea	Morphometrics (FL, LD1, WFW) and reproduction (S, GTW)	ORSTOM	ORSTOM	Albaret (1976)
	IRD_1983-1988	1983-1988	4,245	Learn more about the reproductive conditions of YFT by examining the gonads and comparing the results with older datasets	Morphometrics (LD1) and reproduction (S, MM, GTW)	ORSTOM	ORSTOM	Bard and Capisano (1991)
	RONDEUR	1998-1999	10,915	Obtain biometric relationships of BET, SKJ and YFT for the construction of juvenile exhaust grids on the seines	Morphometrics (TL, FL, LD1, BH, BW, TG0, WFW)	-	IRD	Gaertner et al. (1999)
	DCF	2009-2016	3,501	Biological collection on tropical tunas (BET, SKJ, YFT) and bycatch species caught by purse seiner fleets	Morphometrics (FL, LD1, CFL, TG1, TG2, WFW, GFW), reproduction (S, MM, GTW) and diet (FSW, ESW, preys) + LW, RVW	EU DG MARE	IRD	Bach et al. (2018)
	RTP_ DEBARQUEMENT	2013-present	11,794	Determine the length-weight relationship on tropical tunas and bycatch species	Morphometrics (FL, LD1, WFW)	EU DG MARE	IRD	-
	EUMAP	2017-present	5,310	Biological collection on tropical tunas (BET, SKJ, YFT) and bycatch species caught by purse seiner fleets	Morphometrics (FL, LD1, CFL, TG1, TG2, WFW, GFW), reproduction (S, MM, GTW) and diet (FSW, ESW, preys) + LW, RVW	EU DG MARE	IRD	Bach et al. (2018)
Indian	BIOMCO	1987-1991	2,734	Determine the length-weight relationship, spawning characteristics and sexual maturity of YFT	Morphometrics (FL, LD1, WFW) and reproduction (S, MM, GTW) + LW	-	ORSTOM and SFA	Karpinski and Hallier (1988), Hassani and Stéquert (1990)
	BIOM_BET	1988-1991	938	Determine the length-weight relationship of BET	Morphometrics (FL, LD1, WFW)	-	ORSTOM	-
	DCF	2003-2016	32,943	Biological collection on tropical tunas (BET, SKJ, YFT) and bycatch species caught by purse seiner fleets	Morphometrics (FL, LD1, TG1, WFW), reproduction (S, MM, GTW) and diet (FSW, ESW, preys) + LW, RVW	EU DG MARE	IRD	Bach et al. (2018)
	IOT_STOMACHS	2005-2008	1,144	Monitor the trophic activity of YFT and BET and identify the evolution of their prey resources	Morphometrics (FL, LD1, TG1, WFW), reproduction (S, MM, GTW) and diet (preys)	EU DG MARE	IRD	Marsac et al. (2006)
	EUMAP	2017-present	6,533	Biological collection on tropical tunas (BET, SKJ, YFT) and bycatch species caught by purse seiner fleets	Morphometrics (TL, FL, LD1, TG1, WFW, GFW), reproduction (S, MM, GTW) and diet (FSW, ESW, preys) + LW, RVW	EU DG MARE	IRD	Bach et al. (2018)

**Table 2.** Data file components.

**File names**	**Description**
METADATA.txt	Metadata file of the dataset
ENVIRONMENT.txt	Records of environmental events (from logbooks and well plans)
SPECIMEN.txt	Records of sampling events
Ocean.txt	List of oceans and seas
Quadrant.txt	List of quadrants created according to the Equator and the Greenwich meridian
Gear.txt	List of fishing gear used by fishing vessels
Landing_site.txt	List of ports where fish can be landed
Fishing_mode.txt	List of fishing mode describing the nature of the association of the fish
Vessel_storage_mode.txt	List of conservation methods used by the vessel
Species.txt	List of species
Project.txt	List of projects for which the fish was sampled
Sex.txt	List of genders
Measuring_device.txt	List of measuring devices that can used during sampling
Macro_maturity_stage.txt	List of macro maturity stages
Stomach_prey_group.txt	List of major taxonomic groups of prey found in the stomach

**Table 3.** Number of fish sampled per species and ocean. EAO: Eastern Atlantic Ocean; WIO: Western Indian Ocean; *group of species

**Family**	**Species**	**Vernacular name**	**EAO**	**WIO**	**Total**
Balistidae	*Balistidae spp.	*Triggerfishes, durgon nei	635		635
	*Canthidermismaculata*	Rough triggerfish	45	93	138
Belonidae	*Ablenneshians*	Flat needlefish		3	3
	*Tylosuruscrocodilus*	Hound needlefish		1	1
Carangidae	*Caranx spp.	*Jacks, crevalles nei	3,283		3,283
	*Caranxcrysos*	Blue runner	1		1
	*Caranxsexfasciatus*	Bigeye trevally		2	2
	*Decapterusmacarellus*	Mackerel scad		42	42
	*Elagatisbipinnulata*	Rainbow runner	631	129	760
	*Seriolarivoliana*	Longfin yellowtail		7	7
	*Uraspissecunda*	Cottonmouth jack		63	63
Carcharhinidae	*Carcharhinusfalciformis*	Silky shark		25	25
Coryphaenidae	*Coryphaenidae spp.	*Dolphinfishes nei	53		53
	*Coryphaenahippurus*	Common dolphinfish	3	105	108
Dasyatidae	*Dasyatisviolacea*	Pelagic stingray		1	1
Echeneidae	*Echeneidae spp.	*Suckerfishes, remoras nei		1	1
	*Echeneisnaucrates*	Live sharksucker		1	1
Ephippidae	*Platax spp.	*Batfishes		28	28
Kyphosidae	*Kyphosuscinerascens*	Blue sea chub		9	9
	*Kyphosusvaigiensis*	Brassy chub		36	36
Lobotidae	*Lobotessurinamensis*	Tripletail		15	15
Monacanthidae	*Aluterusmonoceros*	Unicorn leatherjacket filefish		32	32
Scombridae	*Acanthocybiumsolandri*	Wahoo	26	38	64
	*Auxisthazard*	Frigate tuna		41	41
	*Auxisthazard and *Auxisrochei*	*Frigate and bullet tunas	1,383		1,383
	*Euthynnusaffinis*	Kawakawa		30	30
	*Euthynnusalletteratus*	Little tunny	855		855
	*Katsuwonuspelamis*	Skipjack tuna	8,566	4,598	13,164
	*Thunnusalalunga*	Albacore	1	10	11
	*Thunnusalbacares*	Yellowfin tuna	15,833	36,388	51,776
	*Thunnusobesus*	Bigeye tuna	4,700	2,579	7,279
Sphyraenidae	*Sphyraenabarracuda*	Great barracuda	4	15	19
	Total		36,019	44,292	80,311

## Footnotes

*1
https://ec.europa.eu/oceans-and-fisheries/policy/common-fisheries-policy-cfp_fr

*2
https://datacollection.jrc.ec.europa.eu/legislation/previous

*3
https://datacollection.jrc.ec.europa.eu/wps

*4
https://www.ob7.ird.fr/en/

*5
https://en.ird.fr
